# Assessing the Quality, Feasibility, and Efficacy of Electronic Patient Platforms Designed to Support Adolescents and Young Adults With Cancer: A Systematic Review Protocol

**DOI:** 10.2196/resprot.6597

**Published:** 2017-01-17

**Authors:** Gemma Pugh, Lisa McCann

**Affiliations:** ^1^ Health Behaviour Research Centre Department of Epidemiology and Public Health University College London London United Kingdom; ^2^ School of Health Sciences Faculty of Health and Medical Science University of Surrey Guildford, Surrey United Kingdom

**Keywords:** adolescent, neoplasms, telemedicine, review

## Abstract

**Background:**

A range of innovative websites, mobile technologies, eHealth and mHealth platforms have emerged to support adolescents and young adults (AYAs) with cancer. Previous reviews have identified these various applications and solutions, but no review has summarized the quality, feasibility, and efficacy of existing patient platforms (inclusive of websites, mobile technologies, mHealth and eHealth platforms) developed specifically for young people with cancer.

**Objective:**

This paper describes the design of a protocol to conduct a review of published studies or reports which describe or report on an existing platform designed specifically for AYAs who have had a cancer diagnosis.

**Methods:**

A search string was developed using a variety of key words and Medical Subject Heading and applied to bibliographic databases. General data (sample characteristics, patient platform development, design and, if applicable, pilot testing outcomes) will be extracted from reports and studies. Drawing on a previously developed coding schematic, the identified patient platforms will be coded for mode of delivery into (1) automated functions, (2) communicative functions, and (3) use of supplementary modes. An adapted version of the Mobile App Rating Scale (MARS) will be used to assess the of quality of each identified patient platform. The methodological quality of included studies will be assessed using the Quality Assessment Criteria for Evaluating Primary Research Papers from a Variety of Fields (QualSyst). Both authors will independently screen eligible studies for final inclusion and will both be responsible for data extraction and appraisal. Data will be synthesized narratively to provide an overview of identified patient platforms.

**Results:**

The review began in October 2016 and is currently in progress. The review paper will be submitted for peer-review and publication in the summer of 2017.

**Conclusions:**

This review will be unique in its focus on assessing, where possible, the quality and efficacy of patient platforms for adolescents and young adults diagnosed with cancer. Results generated from this review will provide an invaluable insight into the utility of modern technology in supporting young people with cancer.

## Introduction

Specifically as a cancer diagnosis and its subsequent treatment may be the first time a young person independently encounters the health care system and is required to learn self-management skills relating to their health and well-being. Each year approximately 14,000 adolescents and young adults (AYAs) are diagnosed with cancer within Europe [1]. Although this number accounts for only a small proportion of the total cancer diagnoses that occur annually, young people with cancer have unique care needs and require tailored support from the point of diagnosis, during treatment, and throughout their lifetime as survivors of cancer [2-4]. AYAs who have had a cancer diagnosis are at increased risk of cancer recurrence, chronic disease, and often face physical, emotional, and social difficulties [5,6]. Specifically, time spent in hospital can disrupt normal social and educational milestones and common treatment-related side-effects such as hair loss, weight gain, scarring, infertility, and amputation can often impact a young person’s body image, trust in health, and self-esteem [6,7]. Following successful cancer treatment more than 60% of AYA survivors of cancer will experience at least 1 long-term chronic health problem as a result of their original diagnosis and treatment [8]. Addressing the specific care needs of young people with cancer has been at the heart of the AYA cancer profession since its inception in the early 1990s [9]. Internationally, efforts and advancements in research, policy, and care are continually being made to ensure young people with cancer receive the specific medical, emotional, and practical support they require during cancer treatment and beyond [1,5,10]. A cancer diagnosis and its subsequent treatment may be the first time a young person independently encounters the health care system and is required to learn self-management skills relating to their health and wellbeing.

Within this context, websites, mobile technologies, and eHealth platforms have emerged as promising and innovative strategies for assisting young people with cancer in accessing information-rich environments and accessing support suitable to their needs [11]. Such technologies offer potential opportunities for AYA survivors of cancer to self-monitor or self-assess their health needs and access peer-to-peer support in a safe environment. Moreover, remote-based health interventions overcome geographical and time-constraint barriers typically faced by health care professionals and researchers attempting to engage this population [12]. A previous review of mobile and tablet-based apps available to young people with cancer identified 7 apps in total [13]. Of these 7 apps, the majority were piloted in proof-of-concept investigations among only a small sample of young people. Despite noted limitations like this coupled with a lack of empirical testing of the identified apps, Wesley and Fizur (2015) concluded that apps were a favorable means of health intervention for young people with cancer due to the positive perceptions of the usability of the apps and their functionalities [13]. This is reflective of previous studies which report high levels of desire for technology-based information resources and self-management tools among AYAs living with or beyond a diagnosis of cancer [11,12].

However, to date there has been no focused effort to fully synthesize and review existing patient platforms (inclusive of websites, mobile technologies, and mHealth and eHealth platforms) developed specifically for young people with cancer. Understanding component features of existing technology platforms is an important step in characterizing the potential utility of technology-based interventions for young people with cancer. Thus, the aim of this review protocol is to outline the staged approach that will be adopted to address this knowledge gap. The objective of the review is to identify, characterize, and fully assess the quality, feasibility, and efficacy of existing patient platforms developed specifically for adolescents and young adults who have had a cancer diagnosis. Such a comprehensive review will provide further important insight into the utility of technology in the health care and support of AYAs with cancer.

## Methods

The methods to be adopted in this literature review are outlined below and follow the standard Preferred Reporting Items for Systematic Review and Meta-Analysis Protocols guidance [14].

### Inclusion Criteria

#### Types of Studies and Reports

Any study or report which describes or reports an existing electronic platform designed specifically for young people aged between 13 and 39 years of age living with or beyond a cancer diagnosis will be included for review.

#### Types of Participants

The age range of 13 to 39 years has been used to reflect United Kingdom and United States of America accepted definitions of young people with cancer [15,16]. Participants of interest are those who fall within this age bracket and are defined as teenagers, adolescents, or young adults living with or beyond cancer. This includes young people who are AYA-aged survivors of a childhood cancer diagnosis. No studies will be excluded based upon participants’ treatment status or position on the cancer care continuum. This aligns with the National Cancer Institute, World Cancer Research Fund, and American Institute of Cancer Research definitions of cancer survivor as anyone who has had cancer from the point of diagnosis onwards [17,18].

#### Types of Interventions

Patient platforms for the purposes of this review will encompass any eHealth, mHealth, or health informatics efforts which apply modern computing and communication methods such as digital technologies for the provision of health care.

#### Types of Outcome Measures

The efficacy of patient platforms piloted as health interventions within randomized controlled trials or quasi-experimental trials will be assessed by extracting data on health outcomes, specifically the magnitude of change in health outcome.

### Exclusion Criteria

Studies or reports will be excluded from the review if they report on platforms developed for young people with comorbid conditions other than cancer or if AYAs with cancer are not the primary focus of the paper (ie, where AYAs with cancer are included as a subsample of AYAs more generally or AYAs with other illness conditions). In addition, studies or reports with insufficient detail on the target population, intervention, and mode of delivery (even after author contact to clarify) will be excluded from the review. Studies investigating patient platforms designed for adult cancer survivors aged 40 years and older and studies where the mean age of the sample is older than 39 years will also be excluded. Likewise, electronic platforms designed for use exclusively by parents or caregivers and health care professionals who work directly with AYA cancer patients will be not be included in the final review.

### Identification and Screening

A literature search for patient platforms developed specifically for or piloted among AYAs who have had a cancer diagnosis will be conducted. The search string outlined below will be applied to bibliographic databases. Where possible, authors of studies selected for review will be contacted to inquire as to whether they know of any additional patient platforms designed specifically for AYAs living with or beyond a diagnosis of cancer, either published or unpublished.

The search strategy includes a range of Medical Subject Headings terms and a range of relevant keywords for the interventions of interest in this review. Grey literature and the reference lists of all included papers and reports will also be reviewed to identify any additional relevant studies or reports.

Search String: Teen* OR Adolesc* OR Young Adult OR Child AND Cancer OR Cancer Survivor AND app OR apps OR application or mobile OR Android OR droid OR iphone OR ios OR blackberry or web OR internet OR portal OR portlet OR microsite OR website OR “web site” OR url OR mhealth OR ehealth OR internet OR online OR digital OR email OR social network OR electronic communication OR e-health OR e-learning OR elearning OR social network OR facebook OR myspace OR virtual world OR short messaging service OR virtual clinic OR computer assisted therapy OR information technology OR electronic communication OR digital divide OR e-mail OR email OR telehealth.

### Evaluation Criteria

Data will be extracted, appraised, and evaluated to allow a comprehensive synthesis of included studies and reports.

#### Data Extraction

General data and information regarding sample characteristics, patient platform development, design, and (if applicable) pilot testing outcomes will be extracted from reports and studies.

#### Coding of Platform Characteristics

Mode of delivery will be coded based upon the coding scheme developed by Webb and colleagues [19]. Automated functions will be classed as either (1) enriched information environment (eg, supplementary materials, testimonials videos, or games), (2) automated tailored feedback based upon individual progress monitoring (eg, comparison to norms, goals, reinforcing messages, or coping messages), or (3) automated follow up messages (eg, reminders, tips, newsletters, and encouragement). Communicative functions will be categorized into (1) access to an advisor to request advice (eg, ask the expert/expert-led discussions or chat functions), (2) scheduled contact with an advisor (eg, emails), and (3) peer-to-peer support access. Supplementary modes of communication will be classified into email, telephone, short messaging service, CD-ROM, and videoconferencing.

#### Assessment of Quality

An adapted version of the Mobile App Rating Scale (MARS) will be used to evaluate each patient platform. The MARS scale will be used to classify and evaluate each platform in 5 areas: engagement, functionality, aesthetics, information quality, and subjective quality [20].

The methodological quality of included studies will be assessed using the Quality Assessment Criteria for Evaluating Primary Research Papers from a Variety of Fields (QualSyst). This tool incorporates scoring systems previously used for assessing qualitative and quantitative research. There are 14 items to assess quantitative studies and 10 items to assess qualitative studies.

#### Assessment of Feasibility and Efficacy

Data and information regarding reported acceptability, compliance, delivery of the intervention, recruitment, and participant retention will be extracted from each of the studies and reports and synthesized in order to gather an overview of the feasibility of each individual patient platform.

### Data Synthesis

Extracted and appraised data will be collated in relevant Excel tables (Microsoft Corp). Synthesis data will be presented narratively in text and summary tables in the review publication.

## Results

The review began in October 2016 and is currently in progress. The review paper will be submitted for peer-review and publication in the summer of 2017.

## Discussion

The application of technology in the supportive care of AYAs is an emerging field of interest [11]. To date, however, there has been no collective effort to fully synthesize the literature within this area or identify key features and functionalities of existing patient platforms for AYAs with cancer. This methodological review of eHealth, mHealth, or health informatics efforts that apply modern computing and communication methods for the provision of health care and information to AYA cancer patients and survivors will allow an invaluable insight into the range of existing patient platforms for young people with cancer. Furthermore, the use of multiple coding frameworks to classify and assess intervention features will allow rigorous assessment of patient platform quality, feasibility, and efficacy. This approach will provide a novel and comprehensive overview of this topical area. The participant inclusion criteria of this review has purposefully been kept broad in order to reflect international variations in AYA cancer age brackets [15,21] and variations in the terminology used to describe AYA cancer patients and cancer survivors [22]. Equally, the intervention inclusion criteria is broad in order to fully capture the wide range of existing patient platforms for this unique population group. This approach to searching existing literature for studies concerning AYA cancer populations has been previously applied within other systematic reviews [23,24]. It is hoped this review will provide an invaluable insight into existing patient platforms and underscore future developments within this field of cancer research.

## Abbreviations

AYA: adolescents and young adults
MARS: Mobile App Rating Scale
QualSyst: Quality Assessment Criteria for Evaluating Primary Research Papers from a Variety of Fields
